# Identification of Diosmin and Flavin Adenine Dinucleotide as Repurposing Treatments for Monkeypox Virus: A Computational Study

**DOI:** 10.3390/ijms231911570

**Published:** 2022-09-30

**Authors:** Thua-Phong Lam, Viet-Hung Tran, Tan Thanh Mai, Nghia Vo-Trong Lai, Bao-Tran Ngoc Dang, Minh-Tri Le, Thanh-Dao Tran, Dieu-Thuong Thi Trinh, Khac-Minh Thai

**Affiliations:** 1 Department of Medicinal Chemistry, Faculty of Pharmacy, University of Medicine and Pharmacy at Ho Chi Minh City, Ho Chi Minh City 700000, Vietnam; 2 Institute of Drug Quality Control Ho Chi Minh City, Ho Chi Minh City 700000, Vietnam; 3 School of Medicine, Vietnam National University, Ho Chi Minh City 700000, Vietnam; 4 Faculty of Traditional Medicine, University of Medicine and Pharmacy at Ho Chi Minh City, Ho Chi Minh City 700000, Vietnam

**Keywords:** monkeypox virus, repurposing treatment, E8 protein, homology modeling, molecular docking, dynamics simulation, MM/GBSA

## Abstract

The World Health Organization declared monkeypox a global public health emergency on 23 July 2022. This disease was caused by the monkeypox virus (MPXV), which was first identified in 1958 in Denmark. The MPXV is a member of the Poxviridae family, the Chordopoxvirinae subfamily, and the genus Orthopoxvirus, which share high similarities with the vaccinia virus (the virus used to produce the smallpox vaccine). For the initial stage of infection, the MPXV needs to attach to the human cell surface glycosaminoglycan (GAG) adhesion molecules using its E8 protein. However, up until now, neither a structure for the MPXV E8 protein nor a specific cure for the MPXV exists. This study aimed to search for small molecules that inhibit the MPXV E8 protein, using computational approaches. In this study, a high-quality three-dimensional structure of the MPXV E8 protein was retrieved by homology modeling using the AlphaFold deep learning server. Subsequent molecular docking and molecular dynamics simulations (MDs) for a cumulative duration of 2.1 microseconds revealed that ZINC003977803 (Diosmin) and ZINC008215434 (Flavin adenine dinucleotide-FAD) could be potential inhibitors against the E8 protein with the MM/GBSA binding free energies of −38.19 ± 9.69 and −35.59 ± 7.65 kcal·mol^−1^, respectively.

## 1. Introduction

On 23 July 2022, the World Health Organization (WHO) announced monkeypox as a worldwide public health emergency [1]. As of 7 September, more than 52,996 laboratory-confirmed cases of monkeypox and 18 deaths have been identified in 102 countries [2]. The symptoms of human monkeypox virus (MPXV) disease are similar to smallpox infection, including fever, headache, rash, and respiratory symptoms, with the death rate around 3–6% in recent times [3].

The MPXV, first identified in 1958 in non-human primates in Denmark, is a member of the *Orthopoxvirus* (OPXV) of the *Poxviridae* family [4]. OPXV has close genetic and antigenic relationships, making infection with one of the members of the genus an effective vaccination against the others [4]. They are large double-stranded DNA viruses with a genome size of around 190 kb that replicate in the cytoplasm of infected cells. An infected cell consists of four types of virions: intracellular mature virus (IMV), intracellular enveloped virus (IEV), cell-associated enveloped virus (CEV), and extracellular enveloped virus (EEV) [5].

Viral entry of poxvirus into cells includes three main steps: attachment, membrane fusion, and core invasion [6]. The MPXV and vaccinia virus (VACV) genomes share a high degree of similarity, suggesting that their entry-fusion steps may be similar [7]. For VACV, the mature virus attachment to the cell surface is facilitated by the four binding proteins D8, A27, A26, and H3 [6]. The ubiquitously expressed glycosaminoglycans (GAGs) [8], heparan sulfate (HS) [9,10], and chondroitin sulfate (CS) [11] are thought to be important cell surface receptors.

The VACV D8 protein, which is the ortholog of the MPXV E8 protein, binds to CS—the most abundant GAG, and is involved in viral entry [6,12]. Likewise, the MPXV E8 is a 32 kDa type 1 membrane protein [13] with human carbonic anhydrases homology (CAH, residues 1–234) in the large N-terminal and an uncharacterized C-terminal domain (residues 235–273) downstream of the CAH domain [14]. The rest of the protein is composed of a single transmembrane domain (TM, residues 274–294) and a short intra-virion tail (residues 295–304). The central positively charged crevice of the CAH domain is a potential binding site of CS [13]. The deletion of the E8 protein severely affects the binding of mature viruses to GAG [9,10,11], suggesting that inhibiting the protein could reduce the viral transmission rate and ameliorate the symptoms of the disease.

Up until now, there are no FDA-approved drugs for the treatment of the MPXV. The only two FDA-approved medications for treating smallpox that demonstrated some inhibitory activity against the MPXV are tecovirimat and brincidofovir [15]. Drug discovery is a tedious, time-consuming, and heavily expensive process that comes with a high failure rate. Computer-aided drug discovery and drug repositioning are the two strategies to overcome these limitations. Since the first cases of COVID-19 infection, some existing drugs such as remdesivir and a ritonavir/lopinavir combination were repurposed and evaluated for their antiviral activities [16]. Remdesivir and molnupiravir were the two most successful examples of drug repositioning strategies for the treatment of COVID-19 [17]. However, to date, the structure of the MPXV E8 protein has not been reported. This research aims to develop a protein structural homology of the MPXV E8 protein and identify small-molecule inhibitors of the MPXV E8 protein from the global approved drug database.

## 2. Results

### 2.1. Protein Homology Modeling Using Deep Learning Approach

**Figure 1:**
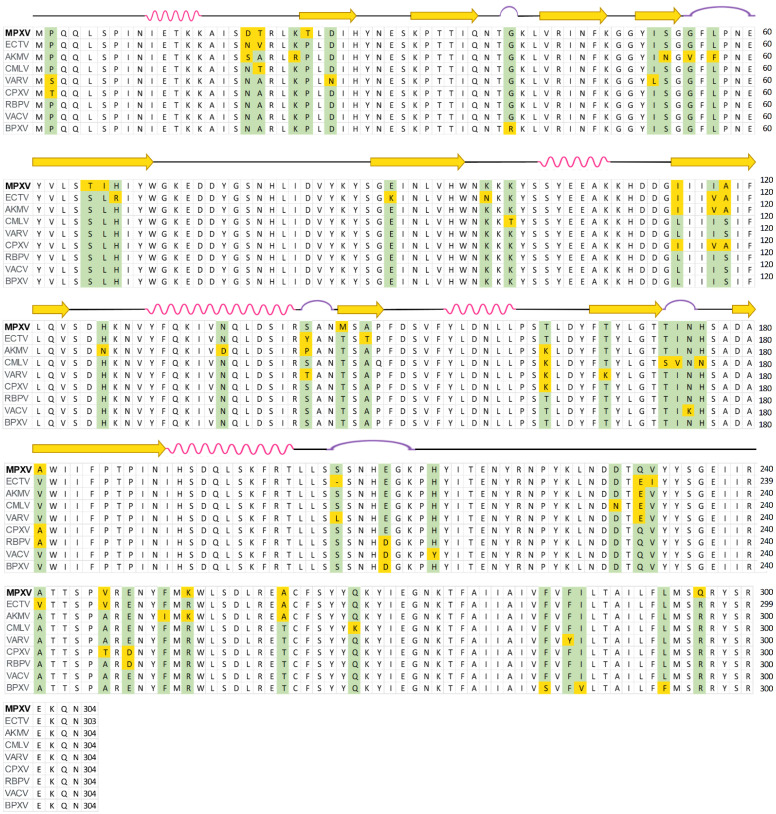
Multiple sequences alignment of the MPXV E8 protein with their orthologs from other members of the Orthopoxvirus genus. The alignment was performed on the Clustal Omega web server (https://www.ebi.ac.uk/Tools/msa/clustalo/) (accessed on 24 July 2022). Green and yellow highlights indicate residue differences between the viruses. Secondary structures are described at the top of the MSA for MPXV E8 protein (yellow arrow: β-sheet; pink loops: α-helices; purple curve: turn). The protein sequences used in the MSA were retrieved from the UniProt server with the corresponding UniProt ID: Monkeypox virus (MPXV-Q8V4Y0); Ectromelia virus (ECTV-Q83439); Akhmeta virus (AKMV-A0A346FRL4); Camelpox virus (CMLV-C9E783); Variola virus (VARV-P0DSY2); Cowpox virus (CPXV-A0A1S5WL42); Rabbitpox virus (RBPV-Q6RZI9); Vaccinia virus (VACV-P20508); Buffalopox virus (BPXV-D0U6N1).

Prior to homology modeling, a quick multiple sequence alignment (MSA) of the MPXV E8 protein with their orthologs from eight *Orthopoxvirus* genus members was performed using the Clustal Omega server (Figure 1). According to the MSA results, the E8 protein is highly conserved across the *Orthopoxvirus* genus. In a side-by-side comparison, the MPXV E8 sequence is substantially similar in the cowpox virus (CPXV), rabbitpox virus (RBPV), and vaccinia virus (VACV), with a Percent Identity of 95.39%, 95.39%, and 94.74%, respectively, spanning the whole 304 residues. The secondary structure of the VACV D8 protein is depicted at the top of the MSA. Because of the lack of structural information about the VACV D8 protein after residue 235, only the structural representation of the first 234 residues were shown [13].

**Figure 2:**
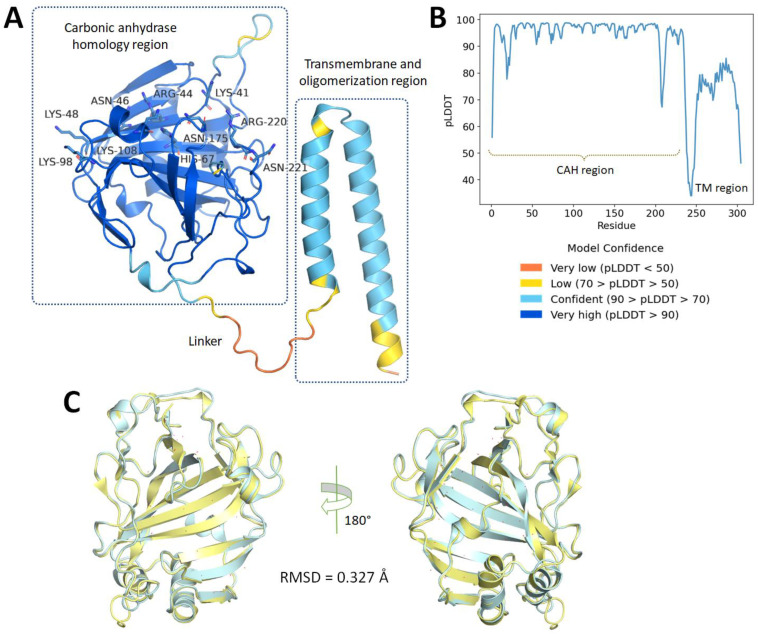
The homology structure of the MPXV E8 protein: (**A**) The model consists of two distinguished regions: (I) Carbonic anhydrase homology (CAH) region, and (II) transmembrane (TM) and oligomerization region. The basic residues that were hypothesized to be a binding pocket of chondroitin sulfate are represented as sticks. (**B**) The predicted local-distance difference test (pLDDT) values over 304 residues of the MPXV E8 protein (the higher the value is, the better confidence the model has). (**C**) The three-dimensional structure of the MPXV E8 protein (yellow cartoon) aligned with the VACV D8 protein (PDB ID: 4E9O) (blue cartoon) with the root mean square deviation (RMSD) value.

Based on the similarity, the MPXV E8 protein homology model was then constructed based on a sequence of 304 amino acids retrieved from the UniProt server (ID: Q8V4Y0) (Figure 1) by the AlphaFold deep learning server (https://alphafold.ebi.ac.uk/) (accessed on 24 July 2022). The model (see Figure 2A) consists of two distinguished domains: domain I (residue 1–235) is a human carbonic anhydrases homology region (CAH), and domain II (residue 249–304) consists of two antiparallel α-helixes. These two domains are connected by a 13 residue-long linkage.

The first domain is a carbonic anhydrase homology (CAH) region, which also plays a role as the ectodomain of this transmembrane protein. Based on the similarity between the MPXV E8 protein and VACV D8 protein (94.74 percent identity over 304 residues as shown in Figure 1), the homology structure would be expected to be substantially comparable to the previously reported structure of the VACV D8 protein (PDB ID: 4E9O) [13]. The homology protein surface is rather hydrophobic, with the surrounding loops and helices covering three-fourths of its surface from the solvent. These β-sheets divide the protein into two distinct halves. A significantly positively charged groove formed by the basic residues Arg220, Asn221, Lys41, Arg44, His67, Asn175, and Lys108 on one side and Asn46, Lys48, and Lys98 on the other side distinguishes the upper half of the protein (Figure 2A). This groove was hypothesized to be a CS binding pocket using an in silico model [13], but no further spectroscopic or co-crystallized structure has been reported to confirm this theory. However, according to the highly negative electrostatic status of CS, which contains both sulfate and carboxylate acid substituents, this binding pocket is the only and most appropriate binding site for CS, leading to subsequent viral entry in the later stage.

The second domain consists of two anti-parallel α-helices that act as a transmembrane, and an oligomerization region for the E8 protein by forming disulfide bonds between Cys262 to promote binding affinity with CS [13]. Until now, no protein structure of this domain has been reported in the literature due to the disorganized and weak electron density signal [13,14]. Because of this and the poor confidence of the AlphaFold server (Figure 2B), this study used only the first CAH domain of the MPXV E8 homology protein (residue 1–235) for later computational screening against approved small-molecule drugs. This domain has high structural similarity with the corresponding domain of the VACV E8 protein with an RMSD value less than 2 Å (Figure 2C).

Furthermore, the validity of the protein was confirmed by the Ramachandran plot (Figure S1), retrieved from the SWISS-MODEL server [18], which indicates a high-quality structure was predicted with 94.85%, and 1.29% in the Ramachandran-favored region, and outliers, respectively.

### 2.2. Identification of Chondroitin Sulfate-E Binding Pocket in the MPXV E8 Protein Using Blind Docking Approach

**Figure 3:**
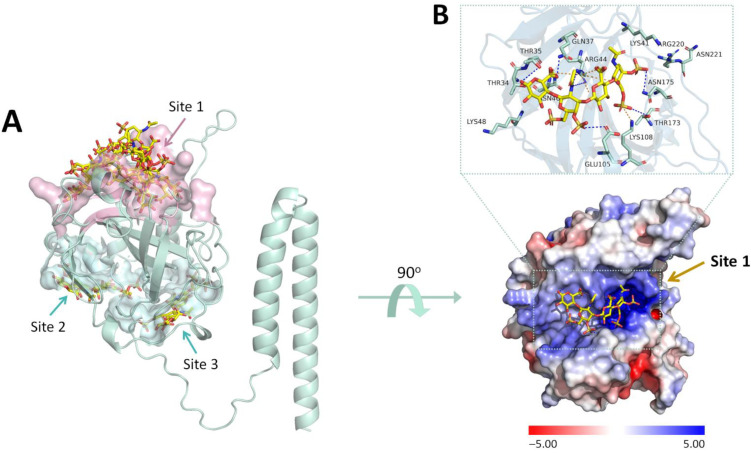
The binding pocket of chondroitin sulfate-E on the MPXV E8 protein: (**A**) The blind docking approach revealed three appropriate binding sites for CS-E, in which seven out of nine ligand conformations were found at Site 1 of the protein (pink). (**B**) The protein surface represents the electrostatic status of the MPXV E8 protein and the binding mode of CS-E in binding site 1. The hydrogen bonds are shown as blue dashes and the salt bridges are shown as orange dashes.

A previous study revealed that the vaccinia virus D8 protein binds selectively to CS-E without interacting with heparan sulfate or other chondroitin sulfation patterns [14]. Applying the blind docking approach with Autodock Vina 1.1.2, the CS-E is also used in this research to re-discover the most likely binding pocket human membrane CS to the E8 protein of the MPXV. As can be seen in Figure 3A, CS-E can bind to three different sites on the protein, in which seven out of nine CS-E conformations were found at Site 1 of the E8 protein. At this binding site, CS-E adheres to the positive charge groove made of the aforementioned basic residues (Figure 3B) with a docking affinity of −6.9 kcal·mol^−1^. The binding pattern was determined to be based on hydrogen bonds and salt bridges between the sulfate or carboxylate substituents and the protein’s basic side chain residues. Based on this binding pattern, a focused docking model was also created to cover all the residues in the basic groove. In short, the grid box center was set to (x = −2.002, y = −10.947, z = −7.038), and the size was set to 20 × 18 × 26 Å. Focused docking using CS-E was also employed to ensure that there was no difference between the blind docking pose and the focused docking pose while employing the same small molecule.

### 2.3. Virtual Screening Using Focused Docking Approach

**Figure 4:**
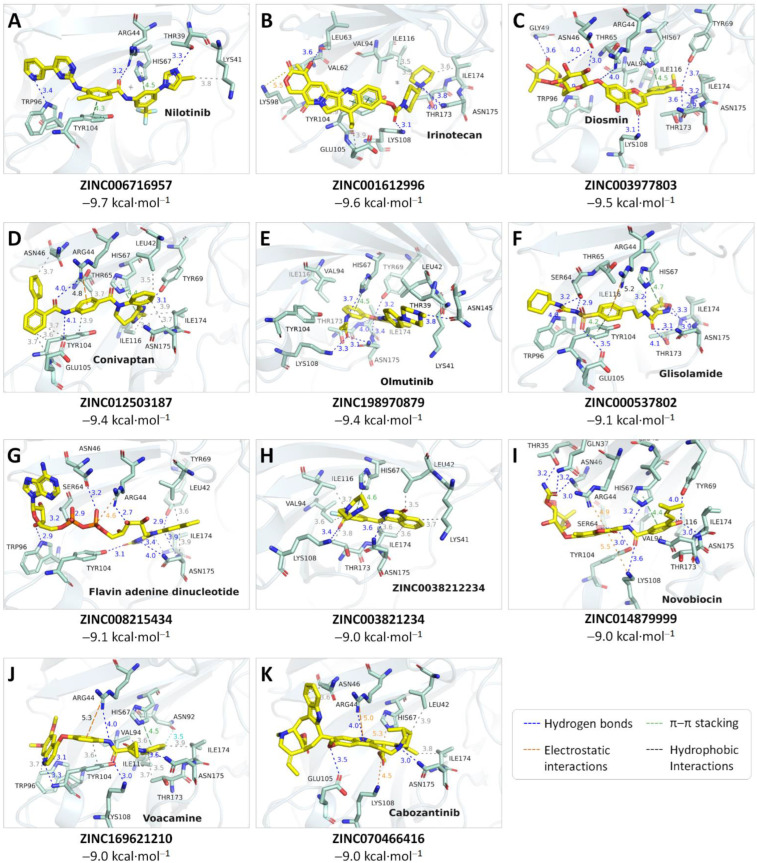
Interactions of the top 11 small-molecule drugs with the MPXV E8 protein obtained by molecular docking. The hydrogen bonds are shown as blue, the π–π stacking (including parallel or perpendicular stacking patterns) is shown as green, the electrostatic interactions (including salt bridges and π–cation interactions) are shown as orange, and the hydrophobic interactions are shown as grey.

Only 5407 of the 5903 small molecules were successfully docked into the binding site of the MPXV E8 protein with negative Autodock Vina docking scores, in which eleven potential compounds were able to form strong interactions with the protein residues with the binding affinity below or equal to −9 kcal·mol^−1^. Based on the hydrophilicity of the binding pocket, the screened compounds were also highly hydrophilic with numerous hydrogen bond donor and acceptor atoms. Their ZINC ID number, international non-proprietary name (INN), docked affinity, and interaction details with the MPXV E8 protein are shown in Figure 4.

### 2.4. Molecular Dynamics Simulations and Binding Free Energy Calculation

Protein–ligand docking provides only static interactions of protein and ligands, as the protein was kept rigid, and the ligand was flexible. To better understand the stability and interactions of top hits and the MPXV E8 protein under physiological conditions, these top hit compounds underwent 100 ns MDs. The most promising compounds could be submitted into long 500 ns MDs to further understand the binding interactions with the E8 protein.

#### 2.4.1. Stability of the Protein-Ligand Complexes

The RMSD, Rg, and SASA values of the protein backbone were used to assess the stability of the protein–ligand complexes. The value of RMSD of the protein backbone and heavy atoms of the ligands during 100 ns simulations are illustrated in Figures S2 and S3, respectively. As can be seen in Table 1, there was no significant difference between the protein backbone either in complex with CS-E or in complex with the hit compounds. Except for the complexes with ZINC001612996 and ZINC003821234, all the other MPXV E8 protein complexes could achieve equilibrium in less than 40 ns and were comparable to each other (Figure S2).

To further investigate the protein stability, the solvent-accessible surface areas (SASA) and radius of gyration (Rg) of the protein backbone were also calculated during the 100 ns MDs duration. In general, an increased value of SASA and Rg indicates the protein unfolding process, therefore reducing the protein stability [19,20]. In agreement with prior RMSD data, there was no substantial variation in the protein backbone in interactions with the hit compounds or their native ligand CS-E.

#### 2.4.2. Ligand Stability and Their Binding Free Energy with the MPXV E8 Protein

**Figure 5:**
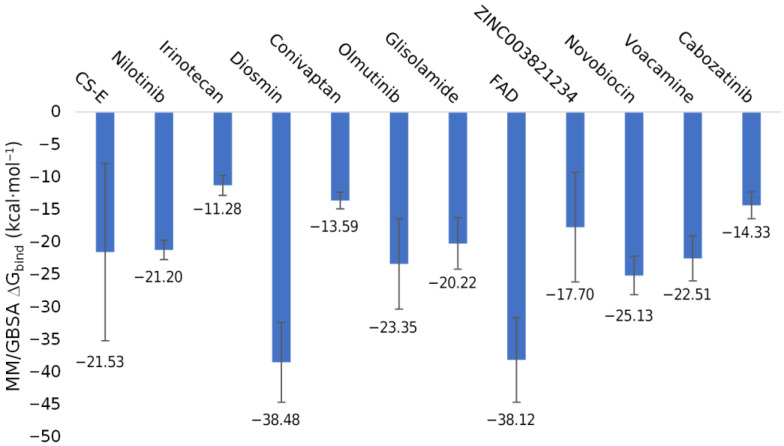
The MM/GBSA binding free energy between chondroitin sulfate-E and the 11 hit compounds with the MPXV E8 protein calculated using the 100 ns MDs trajectories data.

**Figure 6:**
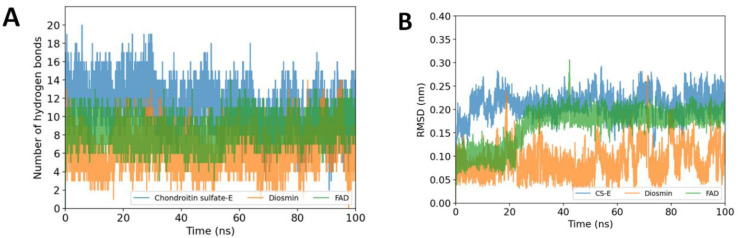
Stability and interactions of the two top hit compounds versus the native ligand CS-E during 100 ns MD simulations: (**A**) The number of hydrogen bonds occurred between the hit compounds and the MPXV E8 protein. (**B**) The RMSD values of ligand heavy atoms.

The binding free energy of the hit compounds in complexes with the MPXV E8 protein was calculated using the MM/GBSA approach. Figure 5 represents the mean values and standard deviations of ∆G_bind_ values throughout the duration of the 100 ns MDs. CS-E interacted with the E8 protein with a ∆G_bind_ of −21.53 ± 13.63 kcal·mol^−1^ despite binding consistently in the binding site by the significantly high occupancy of hydrogen bonds (Figure 6A). In this study, we discovered that ZINC0039778803 (Diosmin) and ZINC008215434 (Flavin adenine dinucleotide—FAD) outperformed CS-E in the MM/GBSA ∆G_bind_ calculation, with the mean value of −38.48 ± 6.15 and −38.12 ± 6.52 kcal·mol^−1^, respectively.

To better understand the binding pattern of CS-E, Diosmin, and FAD during the simulations, the RMSD values of the heavy atoms of the ligands, and hydrogen bond occupancy throughout the simulations were also plotted in Figure 6. Thanks to the abundance of electronegative atoms such as hydroxy groups from sugar substituents (in the case of Diosmin) and diphosphate groups (in the case of FAD), these small-molecule drugs can bind to the E8 protein with high hydrogen bonds occupancy. Throughout the simulations, this characteristic is strikingly comparable to the CS-E binding pattern, which may help to explain the high binding affinity of the hit compounds with the E8 protein. These two compounds were the most promising candidates and were submitted to 500 ns MDs for better evaluation.

### 2.5. Diosmin and FAD as Potential MPXV E8 Protein Inhibitors Proposed by Long Molecular Dynamics Simulations

**Figure 7:**
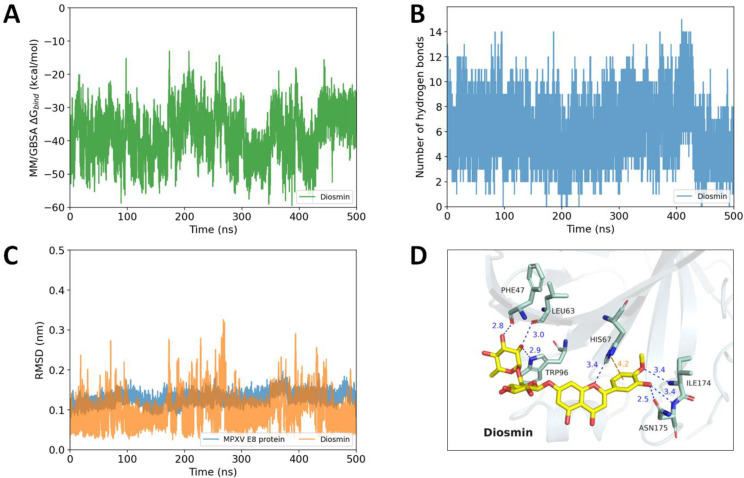
Binding features of Diosmin during 500 ns molecular dynamics simulations: (**A**) The MM/GBSA binding free energy of Diosmin during the simulation. (**B**) The number of hydrogen bonds occurred between Diosmin and the MPXV E8 protein. (**C**) The RMSD values of the MPXV E8 protein (in blue) and the RMSD values of Diosmin heavy atoms (in orange). (**D**) The representative pose of Diosmin in the last 10 nanoseconds of the simulation (hydrogen bonds are shown as blue; π–cation interaction is shown as orange).

During the 500 ns MD simulations, Diosmin was stably bound to the aforementioned binding groove with a MM/GBSA ∆G_bind_ of −38.19 ± 9.69 kcal·mol^−1^ (Figure 7A) and was able to establish at most 15 hydrogen bonds (Figure 7B) with the residues of the binding pocket. The binding affinity during the 500 ns MDs of Diosmin to the MPXV E8 protein remained unchanged in comparison with the first 100 ns MDs. The RMSD plot of the carbon backbone of the MPXV E8 protein and the heavy atoms of Diosmin also revealed a stable binding feature between Diosmin and the E8 protein (Figure 7C). To better understand the binding pattern of Diosmin, the representative pose during the last 10 nanoseconds of the simulation was extracted and submitted to the PLIP server (https://plip-tool.biotec.tu-dresden.de/) (accessed on 30 July 2022) for further analysis. The result showed that Diosmin interacted with the E8 protein by seven hydrogen bonds, in which Phe47, Leu63, His67, Trp96, Ile174, and Asn175 may contribute as hydrogen bond donors or acceptors. Additionally, the aromatic ring B of the Diosmetin (the aglycone structure of Diosmin) could form a π–cation interaction with His67 to strengthen the binding affinity. These features may explain the strong interaction between Diosmin and the MPXV E8 protein (Figure 7D).

**Figure 8:**
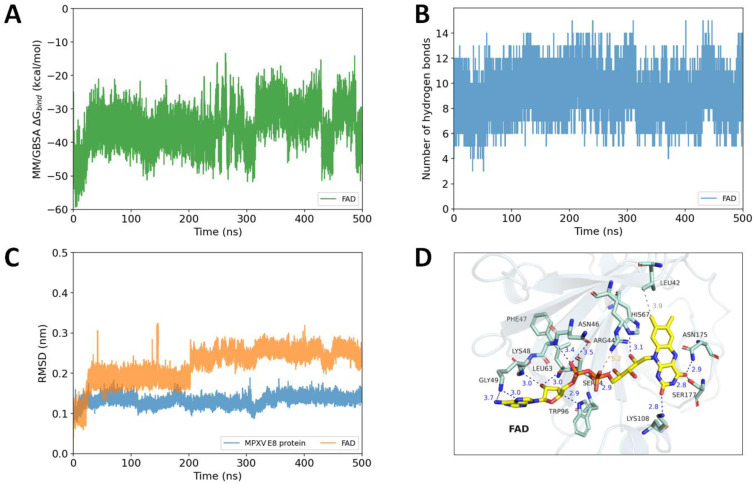
Binding features of FAD during 500 ns molecular dynamics simulation: (**A**) The MM/GBSA binding free energy of FAD during the simulation. (**B**) The number of hydrogen bonds occurred between FAD and the MPXV E8 protein. (**C**) The RMSD values of the MPXV E8 protein (in blue) and the RMSD values of FAD heavy atoms (in orange). (**D**) The representative pose of FAD in the last 10 nanoseconds of the simulation (hydrogen bonds are shown as blue, salt bridge is shown as orange, and hydrophobic interactions are shown as grey).

FAD (flavin adenine dinucleotide) is another promising hit compound against the E8 protein. As was previously mentioned, the diphosphate group and guanidine sidechain of the arginine residue produce salt bridges that serve as the foundation for the binding pattern. This binding property persisted throughout the MD simulations, with the RMSD values of heavy atoms of the ligand fluctuating steadily at about 0.25 nm from 200 ns. In the simulation, FAD could form more hydrogen bonds than Diosmin, with the value varying between 4 and 15 bonds. In addition, the MM/GBSA ∆G_bind_ of −35.59 ± 7.65 kcal·mol^−1^ also outperformed the natural ligand of the MPXV E8 protein, indicating the robust interaction occurred between FAD and the E8 protein. Figure 8 demonstrates that FAD could form up to 12 hydrogen bonds with the protein residues, including Asn46, Phe57, Lys48, Gly49, Leu63, Ser64, His67, Trp96, Lys108, Asn175, and Ser177. The salt bridge between the diphosphate group of FAD and Arg44 previously described in the molecular docking result was also observed, and this binding motif is highly similar to that of CS-E.

## 3. Discussion

The E8 protein is one of the cell surface binding receptors that the MPXV has to attach to the glycosaminoglycan on the surface of human cells, before viral entrance. However, as far as our knowledge, the structure of this protein has not been reported. In this work, a high-quality three-dimensional structure of the E8 protein was built using a deep learning approach on the AlphaFold server. AlphaFold is a computational protein structure prediction program that shows a substantial improvement in modeling accuracy, and can nearly achieve the accuracy expected from experimental methods. In our study, the rebuilt homology model was the fundamental component that was used in subsequent structure-based drug discovery.

Drug repurposing is a strategy for discovering new indications or new use for known approved and investigational drugs. The repurposing candidates have often been through several stages of clinical development before being approved; therefore, their pharmacokinetic and toxicity profiles are well-known [21]. In this study, we found that Diosmin and FAD could be potential candidates against the MPXV E8 protein using computational methodologies.

Diosmin (diosmetin-7-O-rutinoside) is a naturally occurring flavone glycoside that can be isolated from a variety of plant sources or semi-synthesized from hesperidin, another natural flavanone glycoside [22]. Diosmin has previously been shown to have antioxidant, anti-inflammatory, anti-cancer, anti-diabetic, and anti-bacterial properties in various types of disease models [23]. Moreover, the use of natural products and flavonoids has also been reported to have an immunomodulatory effect during the COVID-19 pandemic, which is helpful in the prevention of cytokine storms [24]. In the recent COVID-19 pandemic, Diosmin was also reported as a multi-targeting agent against SARS-CoV-2 [25]. In this study, we would like to propose Diosmin as a potential inhibitor for the MPXV E8 protein, which could help to limit viral entry and inhibit the virus in future fusion stages.

Another promising candidate that we would like to propose is FAD. FAD is the coenzyme form of riboflavin (vitamin B_2_) and has been indicated for various clinical conditions associated with vitamin B_2_ deficiency. In this study, we found that FAD bound consistently to the binding pocket of the E8 protein. Studies have shown that the intracellular reduction–oxidation state plays a vital role in inhibiting viral replication. A previous study showed that FAD could enhance the antiviral activity of interferon-alpha-2a against the influenza virus type A and Herpes simplex virus 1 [26]. Other studies have also suggested FAD as an anti-SARS-CoV-2 agent by interacting with its spike proteins [27], 3-chymotrypsin-like protease [28], and papain-like protease (PLpro) [29]. Using both computational and experimental methods, FAD was proved to have inhibitory activity against the SARS-CoV-2 PLPro with the IC_50_ of 12.39 μM [29]. Another study showed the lung protectability of FAD in H5N1 virus-induced lung injury [30]. In conclusion, our results coupled with evidence from the literature indicate that FAD may play a vital role in the treatment of various types of viruses, via direct viral inhibition as well as indirectly affecting the host immune system.

Although the MPXV was first discovered in the 1960s of the previous century, the specific treatment for this disease remains undeveloped. The smallpox vaccine (JYNNEOS) and some smallpox small-molecule inhibitors (including tecovirimat and brincidofovir) were first developed to combat smallpox eradication and could be potential monkeypox therapeutics [15]. However, as far as we know, the current study is the first computational effort to facilitate the development of novel therapeutics against the MPXV. In the combination with the antiviral potential of these two candidates described earlier, further experimental and clinical trials are required to confirm our results.

## 4. Materials and Methods

### 4.1. MPXV E8 Protein Homology Modeling

The amino acid sequence encoding the MPXV E8 protein (gene ID: E8L) was obtained from the UniProt server (ID: Q8V4Y0) on 24 July 2022. The sequence was then uploaded to the AlphaFold [31] (Google Colab version, DeepMind, London, UK) to construct a homology model. AlphaFold is a novel deep learning system that can predict an unknown protein structure with high quality using both physical and biological knowledge.

To evaluate the protein quality, AlphaFold provides a per-residue confidence score called predicted local-distance difference test (pLDDT). A pLDDT value higher than 90 indicates high confidence and a value lower than 50 indicates low confidence. Additionally, the Phi/Psi torsion angles of all protein residues were plotted in the Ramachandran map using SWISS-MODEL (SIB, Lausanne, Switzerland) program [18]. A high-quality model would be expected to have over 90% in the core regions, also called the favored regions.

### 4.2. Preparation of the Target Protein

Following the validation stage, the protein was prepared by the AutoDock Tools 1.5.7 (The Scripps Research Institute, La Jolla, CA, USA) program [32], in which the protein polar hydrogen atoms and the Kollman charges were added. The structure was then converted to *.pdbqt format and was ready for the subsequent molecular docking procedure.

### 4.3. Preparation of Small-Molecule Ligands

In this study, the ZINC20 (https://zinc20.docking.org/) (accessed on 30 July 2022) “world” subset comprising 5903 small molecule structures approved globally [33] was employed. The downloaded structures were then energy minimized and converted into *.pdbqt format using the OpenBabel 2.4.1 (Pittsburgh, PA, USA) software [34]. The energy minimization process was performed using the MMFF94 forcefield with the steepest descent algorithm. During the energy minimization step, the protonation stage of the small molecules was also calculated employing the same forcefield. The structure of chondroitin sulfate E (CS-E—chondroitin-4,6-disulfate) was built based on the hexamer chondroitin-4-sulfate (CS) ligand retrieved from CS/CathepsinK complex structure (PDB ID: 3C9E) [35]. Additional sulfate groups were added to the ligand using the PyMOL 2.5.2 (Schrödinger, NY, USA) software [36] and then subsequently energy was minimized using the OpenBabel program.

### 4.4. Virtual Screening Based on Blind Docking and Focused Docking

The molecular docking process was performed using the AutoDock Vina 1.1.2 (The Scripps Research Institute, La Jolla, CA, USA program [37], in which the docking process was divided into two stages: (1) blind docking to determine the most likely binding pocket of CS-E, and (2) focused docking for the virtual screening purpose. To explore all possible binding pockets, the exhaustiveness value of each docking stage was set to 128 and 16, respectively. The hit candidates were selected based on their Autodock Vina binding affinity (in kcal·mol^−1^) and protein–ligand binding pattern, including hydrogen bonds, salt bridges, hydrophobic interactions, and π–cation interactions using the Protein–ligand Interaction Profiler (PLIP) server [38].

### 4.5. Molecular Dynamics Simulations

Molecular dynamics simulations (MDs) were carried out using the Gromacs 2021.4 (Royal Institute of Technology and Uppsala University, Sweden) package [39] and the CHARMM-27 (University of Maryland, Baltimore, MD, USA) forcefield. Parameters for the ligand were generated using the SwissParam (SIB, Lausanne, Switzerland) server [40]. This study used the protocol that was previously described by Justin A. Lemkul [41]. Briefly, our simulation process used a dodecahedron box with an edge of 1 nm, in which the system was solvated in the TIP3P water model and neutralized by adding Na^+^ or Cl^−^ ions. The system was then energy minimized for at most 50,000 steps using steepest descent minimization until the maximum force was less than 10 kJ.mol^−1^ to avoid any spatial conflicts or geometrical incompatibilities. The following equilibration was conducted in two 100 ps equilibrium phases, namely, NVT and NPT ensembles. During the equilibration, the temperature was increased to 300 K in the NVT step using the V-rescale thermostat [42] and the pressure was stabilized at 1 bar in the NPT step using the Parrinello–Rahman pressurization unit [43]. After achieving equilibrium, the MDs for each complex were produced in 100 ns or 500 ns at 300 K temperature and 1 bar pressure.

The MD simulations results were saved every 0.01 ns and will be interpreted using the VMD [44] and the PyMOL software [36]. During the simulations, the hydrogen bonds could form between the small-molecule ligands and the MPXV E8 protein residues. In this study, the hydrogen bonds were defined to occur if the bonding angle between the hydrogen donor (D) and acceptor (A) D-H⋯A larger than 120° with the distance between D and A not exceeding 3.5 Å [45]. The GROMACS prebuilt commands were used to calculate the root mean square deviation (RMSD), radius of gyration (Rg), and solvent-accessible surface area (SASA) of the protein backbone or the heavy atoms of the ligands for further evaluation.

### 4.6. End-State Binding Free Energy Calculation

The gmx_MMPBSA package [46] was used to estimate the end-state binding free energy (∆G_bind_) of the ligand–protein complexes. In this study, the Molecular mechanics/Generalized Born surface area (MM/GBSA) method was utilized for the binding free energy. The ∆G_bind_ is defined as the change between the free energy of the protein–ligand complex and that of each component in aqueous solvation (Formula (1). The free energy of each component was based on the changes in the gas phase molecular mechanics energy (E_MM_), solvation free energy (G_solvate_), and conformational entropy (−TΔS) upon the ligand binding (Formula (2). The salt concentration, the temperature, and the solvent model were set to 0.15 M, 298.15 K, and GB-OBC2 [47], respectively. The calculation was conducted throughout the trajectories with the frame interval set to 5, leading to the aggregation of 2000 and 10,000 frames for each of the 100 ns and 500 ns MD simulations, respectively.

ΔG_bind_ = G_complex_ − (G_protein_ + G_ligand_) (1)

Gx = E_MM_ + G_solvate_ − TΔS (2)

## 5. Conclusions

The WHO declared monkeypox a global public health emergency on 23 July 2022, and virtual screening has been a time and money-saving approach to discover a new treatment for a novel disease. In this study, we have targeted the E8 protein of the monkeypox virus, which is vital for the attachment of the virus to the chondroitin sulfate polysaccharide located on the membrane surface of human cells. Using deep learning-based homology modeling, molecular docking, molecular dynamics simulation, and binding free energy calculation, the present study suggests that Diosmin (∆G_bind_ of −38.19 ± 9.69 kcal·mol^−1^)_,_ and FAD (∆G_bind_ of −35.59 ± 7.65 kcal·mol^−1^) could be potential treatments against the E8 protein of the virus, which could decrease the transmission rate of this virus. Further experimental studies should be conducted to validate the current results.

## Figures and Tables

**Table 1:** The mean and standard deviation of protein backbone RMSD, solvent accessible surface area (SASA), radius of gyration (Rg), and ligand RMSD values calculated from the data of 100 ns MDs trajectories of the MPXV E8 protein in complex with CS-E and the 11 hit compounds.

ZINC ID	INN	MPXV E8 Protein Backbone			Ligand Heavy Atoms
		RMSD (nm)	SASA (nm^2^)	Rg (nm)	RMSD (nm)
Chondroitin sulfate-E		0.132 ± 0.015	124.882 ± 1.071	1.737 ± 0.006	0.211 ± 0.025
ZINC006716957	Nilotinib	0.117 ± 0.011	124.921 ± 0.991	1.743 ± 0.006	0.190 ± 0.034
ZINC001612996	Irinotecan	0.149 ± 0.036	125.139 ± 1.295	1.743 ± 0.012	0.095 ± 0.029
ZINC003977803	Diosmin	0.119 ± 0.012	124.627 ± 1.043	1.731 ± 0.005	0.090 ± 0.032
ZINC012503187	Conivaptan	0.126 ± 0.014	125.343 ± 0.967	1.742 ± 0.007	0.194 ± 0.045
ZINC198970879	Olmutinib	0.125 ± 0.015	125.203 ± 1.221	1.739 ± 0.007	0.290 ± 0.086
ZINC000537802	Glisolamide	0.140 ± 0.017	124.362 ± 0.997	1.737 ± 0.006	0.193 ± 0.034
ZINC008215434	FAD	0.131 ± 0.014	124.941 ± 1.020	1.743 ± 0.007	0.166 ± 0.039
ZINC003821234	Unnamed	0.146 ± 0.028	124.595 ± 1.124	1.742 ± 0.006	0.196 ± 0.056
ZINC014879999	Novobiocin	0.105 ± 0.011	124.598 ± 0.981	1.741 ± 0.006	0.212 ± 0.081
ZINC070466416	Cabozantinib	0.126 ± 0.016	125.367 ± 0.992	1.743 ± 0.007	0.203 ± 0.045
ZINC169621210	Voacamine	0.167 ± 0.039	124.785 ± 1.036	1.740 ± 0.006	0.076 ± 0.020

## Data Availability

Not applicable.

## Supplementary Materials

The following supporting information can be downloaded at: https://www.mdpi.com/article/10.3390/ijms231911570/s1.

## Abbreviations

CAH	Carbonic anhydrase homology
CS-E	Chondroitin sulfate-E
FAD	Flavin adenine dinucleotide
GAG	Glycosaminoglycan
MPXV	Monkeypox virus
MSA	Multiple sequence alignment
Rg	Radius of gyration
RMSD	Root mean square deviation
SASA	Solvent accessible surface area
MDs	Molecular dynamics simulation
VACV	Vaccinia virus
WHO	World Health Organization
MM/GBSA	Molecular mechanics/Generalized Born Surface Area
FDA	Food and Drug Administration
