# Supplementary material for: Identification of Diosmin and Flavin Adenine Dinucleotide as Repurposing Treatments for Monkeypox Virus: A Computational Study
Source: Int J Mol Sci. 2022 Sep 30;23(19):11570. doi: 10.3390/ijms231911570 (PMC9570275; doi:10.3390/ijms231911570)
Supplement: Supplementary file 1 [file ijms-23-11570-s001.zip › ijms-1934545-supplementary.pdf]

Identification of Diosmin and Flavin Adenine Dinucleotide as Repurposing Treatments for Monkeypox Virus: A Computational Study

Thua-Phong Lam [1,†], Viet-Hung Tran [2,*,†], Tan Thanh Mai [1], Nghia Vo-Trong Lai [1], Bao-Tran Ngoc Dang [1], Minh-Tri Le [1,3], Thanh-Dao Tran [1], Dieu-Thuong Thi Trinh [4] and Khac-Minh Thai [1,*]

1 Department of Medicinal Chemistry, Faculty of Pharmacy, University of Medicine and Pharmacy at Ho Chi Minh City, Ho Chi Minh City 700000, Vietnam
2 Institute of Drug Quality Control Ho Chi Minh City, Ho Chi Minh City 700000, Vietnam
3 School of Medicine, Vietnam National University, Ho Chi Minh City 700000, Vietnam
4 Faculty of Traditional Medicine, University of Medicine and Pharmacy at Ho Chi Minh City, Ho Chi Minh City 700000, Vietnam
* Correspondence: tran.viethung168@gmail.com (V.-H.T.); thaikhacminh@ump.edu.vn (K.-M.T.)
† These authors contributed equally to this work.

Figure S1. Ramachandran plot of the first domain of the MPXV E8 protein (residues 1–235) 2

Figure S2. Protein RMSD plots (carbon backbone) of 11 complexes of the MPXV E8 protein with the top 11 small-molecule drugs during the 100 nanoseconds molecular dynamics simulations 4

Figure S3. RMSD plots of ligand heavy atoms in 11 complexes with the MPXV E8 protein during the 100 nanoseconds molecular dynamics simulations 6

Abbreviations:

CS-E: chondroitin sulfate-E

MPXV: monkeypox virus

RMSD: Root mean square deviation

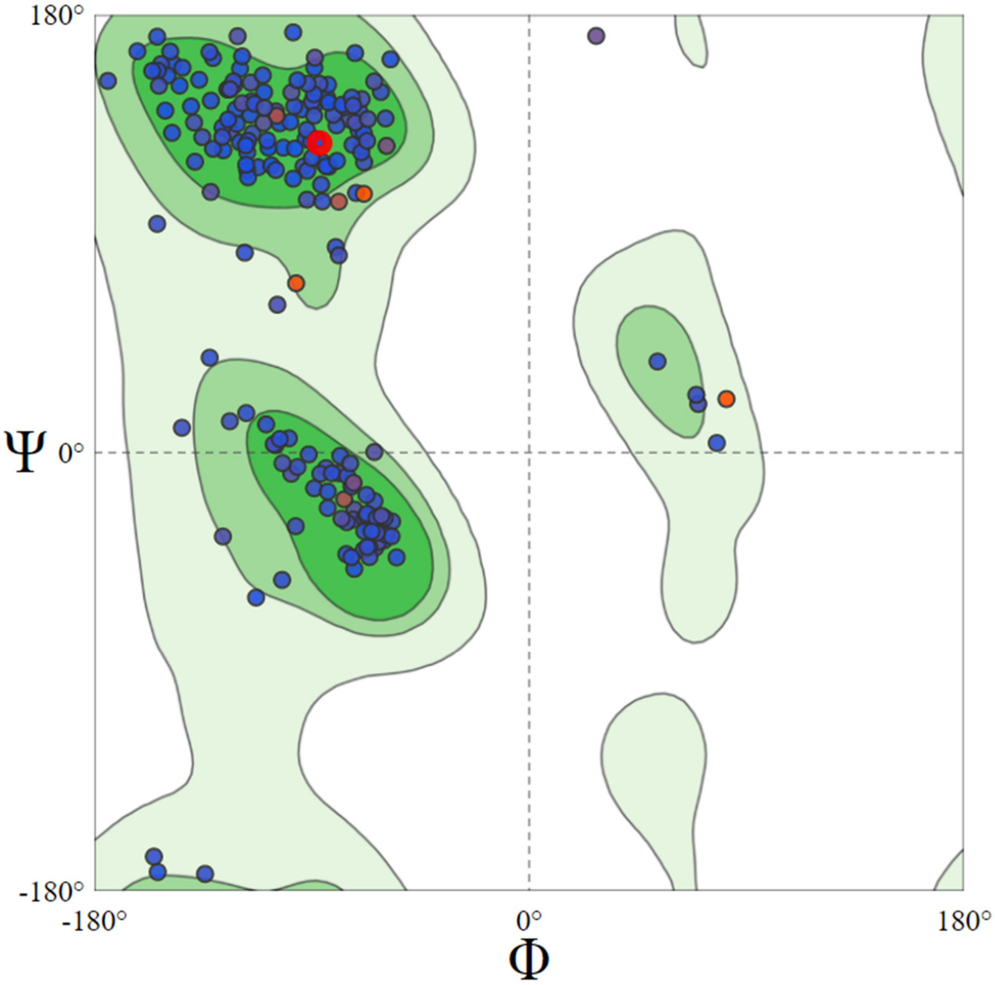

Figure S1. Ramachandran plot of the MPXV E8 protein first domain (residue 1–235)

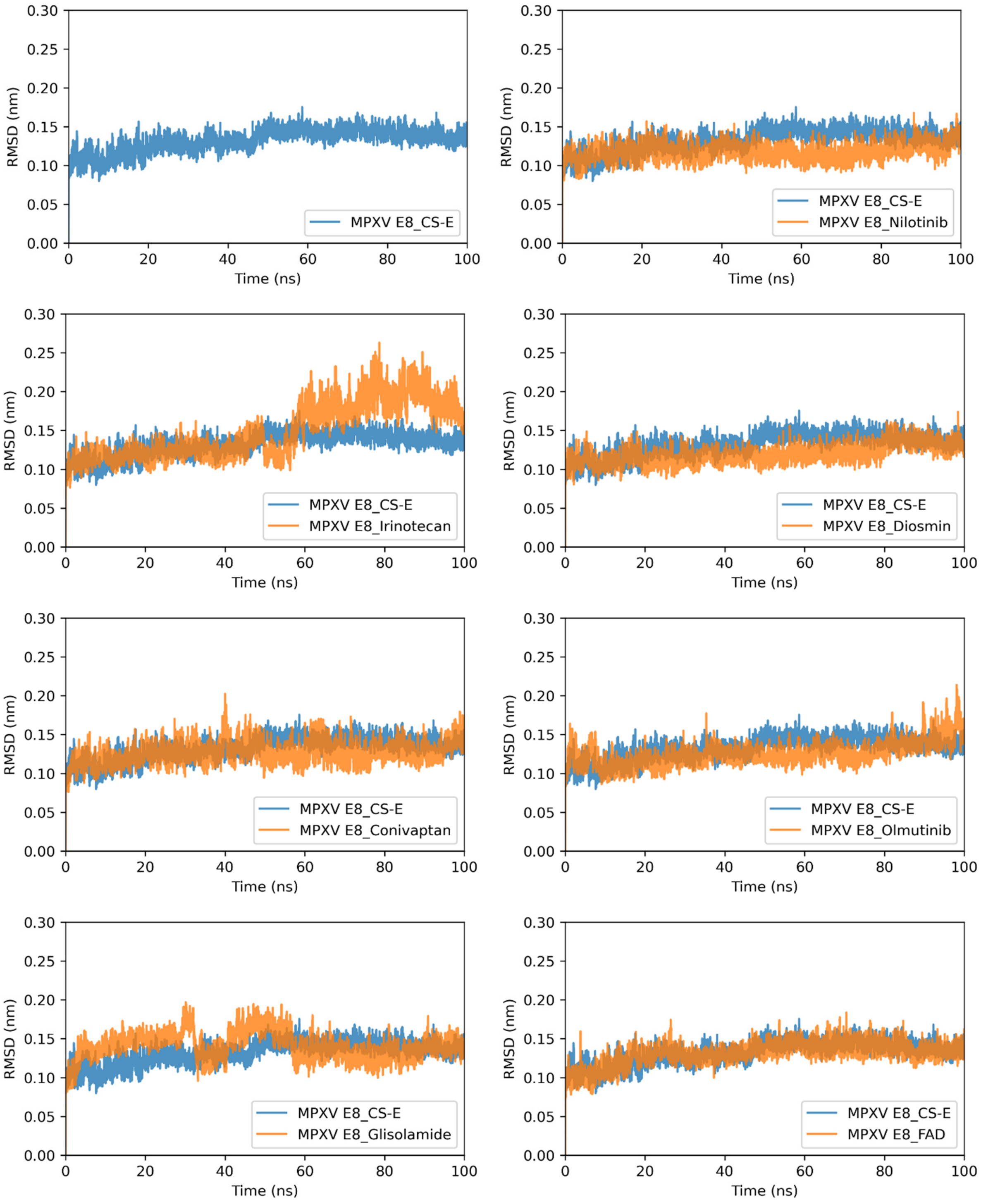
RMSD (nm)
Time (ns)
0.00
0.05
0.10
0.15
0.20
0.25
0.30
0
20
40
60
80
100
MPXV E8_CS-E
MPXV E8_Nilotinib
MPXV E8_Irinotecan
MPXV E8_Diosmin
MPXV E8_Conivaptan
MPXV E8_Olmutinib
MPXV E8_Glisolamide
MPXV E8_FAD

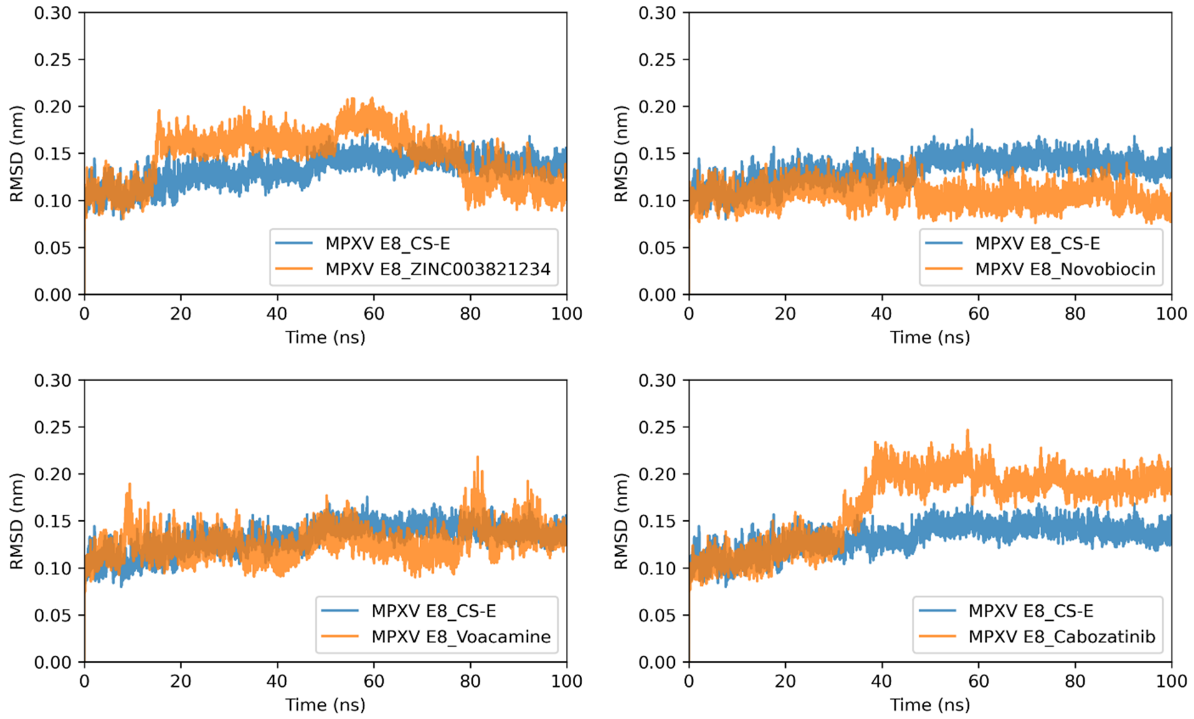

Figure S2. Protein RMSD plot (carbon backbone) of 11 complexes of the MPXV E8 protein with the top 11 small-molecule drugs during the 100 nanoseconds molecular dynamics simulations

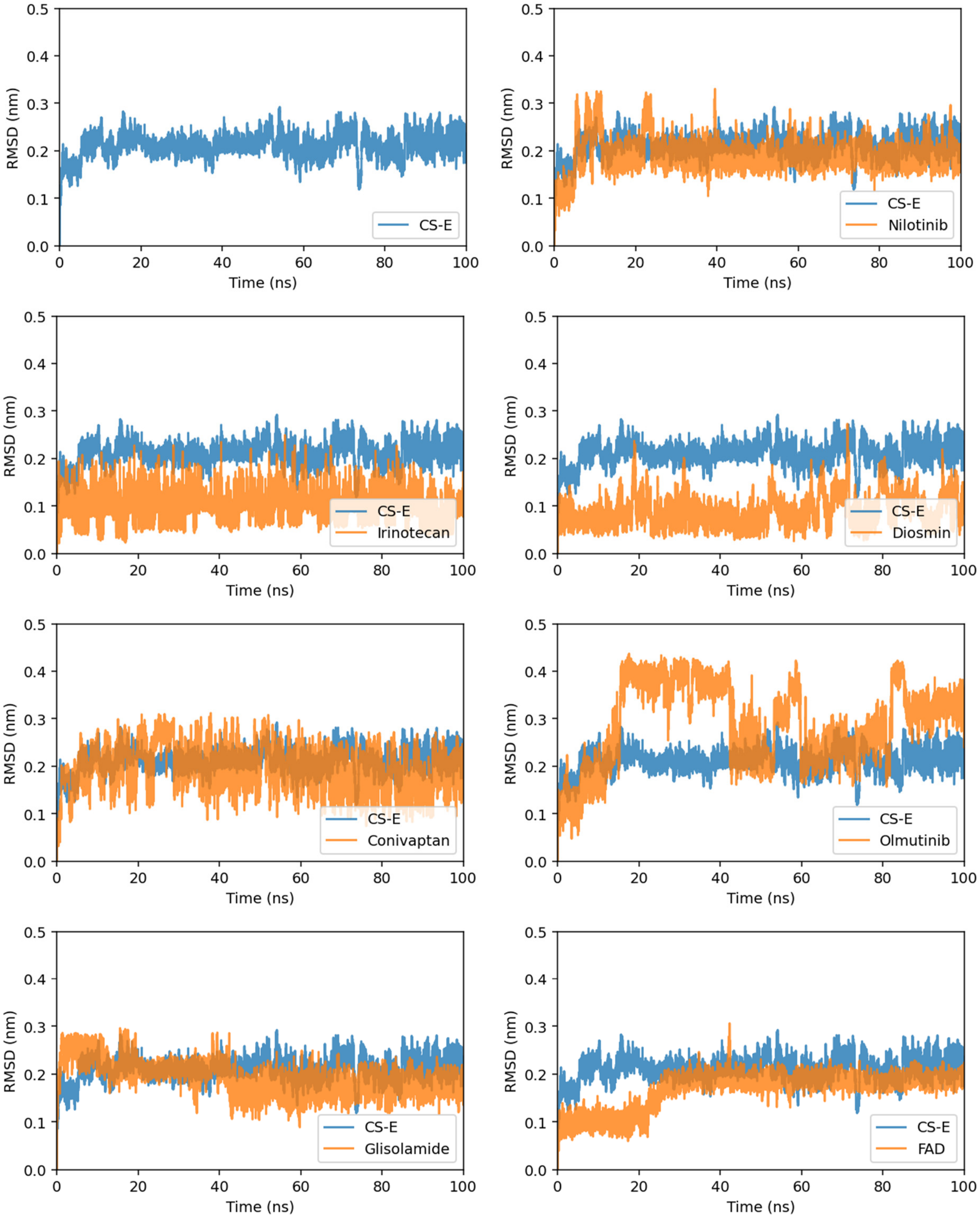
RMSD (nm)
Time (ns)
CS-E
Nilotinib
Irinotecan
Diosmin
Conivaptan
Olmutinib
Glisolamide
FAD

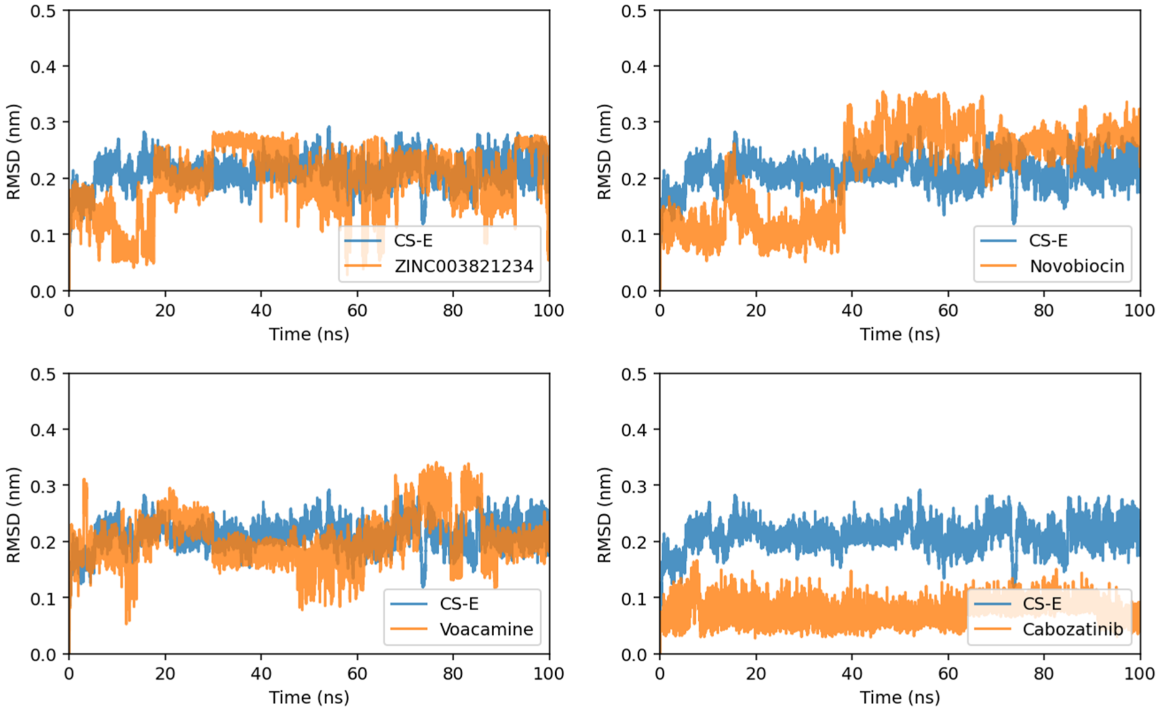

Figure S3. RMSD plot of ligand heavy atoms in 11 complexes with the MPXV E8 protein during the 100 nanoseconds molecular dynamics simulations
